# Placental Health Score (PHS) for Early Prediction of Placental Insufficiency and Preterm Birth: A Cross-Sectional Analytical Study

**DOI:** 10.7759/cureus.94521

**Published:** 2025-10-14

**Authors:** Mamata Panigrahi, Shweta Solan, Prajnaparamita Samanta

**Affiliations:** 1 Anatomy, Kalinga Institute of Medical Sciences, Bhubaneswar, IND

**Keywords:** fetal growth retardation, morphometric analysis, neonatal outcomes, placental health score, placental insufficiency, placental morphology, preterm birth

## Abstract

Background

Placental insufficiency is a pathological state in which the placenta fails to provide adequate oxygen and nutrients to the fetus, leading to impaired growth, preterm birth, and adverse neonatal outcomes. Despite its clinical relevance, diagnostic approaches remain limited, and there is a need for a quantifiable tool to assess placental health. The objective of this study was to develop a Placental Health Score (PHS) by integrating key morphometric placental parameters into a multiple linear regression model after normalization and to validate the score by assessing its correlation with gestational age and neonatal outcomes, particularly the five-minute Apgar score.

Materials and methods

A cross-sectional, analytical study was carried out over two years, from 2022 to 2024, on 50 placentas: 36 were preterm and 14 were term placentas, derived from singletons without any maternal comorbidity. Eleven morphometric parameters were assessed: placental weight, central thickness, peripheral thickness, circumference, fetal weight, birth-weight-to-placental-weight ratio (BW:PW), number of cotyledons, umbilical cord length, umbilical cord diameter, umbilical cord insertion type, and presence of knots. Placentas were collected with informed consent from the participants and approval from the Institutional Ethics Committee and the Departments of Obstetrics and Gynecology. To maintain homogeneity in the study population, pregnancies complicated by maternal comorbidities, multiple gestations, or fetal anomalies were excluded. Statistical analyses included correlation testing and regression modeling, performed with appropriate significance thresholds.

Results

Of the 11 morphometric placental parameters analyzed, five were selected for inclusion in the PHS based on their statistical association with gestational age and their established biological relevance. Central placental thickness (Spearman r = 0.64, 95% CI: 0.37 to 0.92, p < 0.001) and fetal weight (r = 0.51, 95% CI: 0.23 to 0.78, p < 0.001) showed the strongest positive correlations with gestational age, reflecting their direct relationship with placental growth and function. Although the birth-weight-to-placental-weight ratio (BW:PW) ratio exhibited a weaker, negative correlation (r = -0.24, p = 0.0913), it was retained due to its clinical importance as a functional index of placental efficiency. Similarly, cotyledon count (r = -0.05, p = 0.7187) and umbilical cord length (r = -0.02, p = 0.8984) were included for their anatomical relevance: cotyledon number reflects villous arborization and structural maturation of the placenta, while cord length is associated with fetal movement and vascular dynamics, both of which are implicated in placental insufficiency and growth restriction. Thus, the final PHS integrates both statistically supported and biologically meaningful parameters, ensuring a composite score that reflects the multifactorial nature of placental development and its role in determining gestational duration.

Conclusion

The PHS, derived from multiple morphometric parameters, provides a reproducible, objective, and clinically translatable measure of placental health. By integrating anatomical and functional indices into a single score, the PHS enables early prediction of placental insufficiency and preterm birth risk, offering a valuable tool for prenatal risk stratification and improved perinatal care.

## Introduction

Preterm birth remains an urgent global public health issue, significantly contributing to neonatal mortality, morbidity, and long-term healthcare burdens. According to the World Health Organization (WHO), preterm birth is defined as childbirth occurring before 37 completed weeks of gestation and affects approximately 15 million neonates annually, accounting for over 10% of all live births globally [1]. Preterm birth complications were responsible for approximately one million deaths in 2015 alone, making it the leading cause of mortality among children under five years of age [2]. The adverse outcomes associated with preterm delivery extend beyond immediate neonatal survival, manifesting as prolonged health challenges. Preterm neonates face significantly elevated risks for respiratory distress syndrome, neurodevelopmental impairments, vision and hearing disorders, cerebral palsy, and increased susceptibility to chronic non-communicable diseases later in life, such as cardiovascular disease and diabetes mellitus [3,4]. These long-term complications, including chronic respiratory illness, neurodevelopmental delay, and increased susceptibility to noncommunicable diseases, not only impact affected individuals and their families but also impose considerable economic strain on healthcare infrastructures, particularly in low- and middle-income countries (LMICs). Geographically, the burden of preterm birth disproportionately affects regions with limited resources, primarily Asia and sub-Saharan Africa, which together account for approximately 81% of global preterm births [5]. India alone exhibits one of the highest national preterm birth rates, estimated at 13%, highlighting both demographic challenges and persistent gaps in maternal healthcare infrastructure [6]. A key factor in the etiology of preterm labor is placental insufficiency-a pathologic state of suboptimal delivery of oxygen and nutrients to the fetus as a result of compromised placental function [7]. Established diagnostic tools, such as ultrasonographic assessment and postpartum histopathologic analysis, although informative, are limited by subjectivity, operator variability, and the retrospective nature of the latter. Placental morphometric analysis shows promise as a useful tool for improving prenatal risk assessment. Placental thickness, placental weight, number of cotyledons, cord length, and cord insertion type provide quantifiable and reproducible markers that correlate strongly with fetal well-being and neonatal outcomes [8].

Despite growing recognition of the contribution of the placenta to fetal advancement, there exists a fundamental shortcut in the availability of objective, composite measures to estimate placental well-being and predict preterm birth. Present approaches are frequently determined by solitary morphometric parameters or by the examination of retrospectively obtained histopathologic results, reducing their potential for real-time use in the clinic. No study has systematically combined multiple criteria for the placenta in a reproducible score that may serve for prenatal risk stratification [9].

In spite of growing recognition of the contribution of the placenta to fetal advancement, there exists a fundamental shortcut in the availability of objective, composite measures to estimate placental well-being and predict preterm birth. Present approaches are frequently determined by solitary morphometric parameters or by the examination of retrospectively obtained histopathologic results, reducing their potential for real-time use in the clinic. No study has systematically combined multiple criteria for the placenta in a reproducible score that may serve for prenatal risk stratification.

Herein, we present the PHS, a new, regression-weighted score that incorporates five important placental parameters-central thickness, fetal weight, BW:PW ratio, the number of cotyledons, and umbilical length-into a single quantitative value. This composite score correlates strongly with gestational age and neonatal outcomes, including Apgar scores, providing a biologically significant and clinician-translatable tool for the early detection of placental insufficiency. The PHS marks a first-in-field attempt to unite anatomical measurement and predictive obstetric practice, and it will demonstrate application in the resource-limited environment in which non-invasive, standard tools are critical. Prior work links individual morphometric traits-such as placental thickness, weight, and cotyledon count-to neonatal outcomes [4].

Yet no comprehensive scoring system combines these into a predictive model for preterm birth.

The objective of this study is to create and validate an objective and sensitive PHS from morphometric placental measurements. This is to achieve a clinically translatable scoring system with the ability to risk-stratify placental insufficiency and preterm birth, ultimately leading to better prenatal care and fetal outcomes.

## Materials and methods

Methodology

We performed a cross-sectional analytical study of 50 singletons' placentas, which were divided into preterm (<37 weeks, n = 36) and term (≥37 weeks, n = 14) deliveries. Institutional Ethical Committee approved the study (Ref. No. KIIT/KIMS/IEC/912/2022). This was a cross-sectional analytical study conducted over a period of two years (2022-2024), after obtaining written approval from the Departments of Obstetrics and Gynaecology. During this period, placentas were collected according to a predefined protocol, following informed consent from women at the time of delivery in the Department of Obstetrics and Gynaecology, and with clearance from the Head of Department. For homogeneity of samples and to control confounding factors, the following criteria of inclusion and exclusion were applied:

The study included women with singleton pregnancies who had no known maternal comorbidities to ensure a uniform and low-risk population. Exclusion criteria were carefully applied to avoid confounding factors, such as twin or multiple pregnancies and the presence of chronic maternal conditions like diabetes or hypertension. This selection strategy was intended to create a homogeneous study group, minimizing variables that could influence outcomes and ensuring that the results reflected the true associations under investigation.

Sample Size

The sample size was calculated a priori to ensure adequate statistical power for detecting meaningful differences. Using Cohen’s criteria for a medium effect size (d = 0.5), with a two-tailed significance level of 5% (α = 0.05) and statistical power of 80% (β = 0.20), the minimum required sample size for a two-group comparison was estimated at 63 cases, based on the formula [10]:

n = (2 × (Z₁₋ₐ/₂ + Z₁₋ᵦ)²) / d²

For correlation analysis in a single-sample design, Cohen’s standard tables and G*Power (Heinrich-Heine-Universität Düsseldorf, Düsseldorf, Germany) calculations indicated that 44 cases would be sufficient under the same assumptions. Considering practical feasibility and to strengthen the robustness of findings, a final sample size of 50 placentas was chosen. This number provided both statistical adequacy and methodological soundness for the study.

Sampling

Participants were recruited consecutively from a tertiary care hospital. A total of 50 placentas were analyzed, comprising 36 from preterm deliveries (<37 weeks) and 14 from term deliveries (≥37 weeks).

Data Collection: Placental and Umbilical Measurements

The specimens exhibited variation in size, shape, thickness, and umbilical cord insertion points. This morphometric diversity aligned with quantitative findings linking placental features, such as thickness, weight, and fetal weight, to gestational age.

Systematic measurements were carried out in a standardized manner [11-13]. A vertical midline and a perpendicular midline at specified peripheral locations were created with a sterile needle to measure central and peripheral placenta thickness. An immediate measurement post-delivery was carried out to capture placenta weight on a digital weight scale; term placentas typically weighed more than 500 grams compared to preterm placentas, which weighed less. Placenta circumference was measured around the maximum diameter with a non-elastic tape.

Fetal weight taken from delivery records was used as an index of placental efficiency, and the birth-weight-to-placental-weight (BW:PW) ratio was subsequently determined. Ratios below five were considered to be suggestive of possible growth restriction. Cotyledons were carefully counted off the chorionic surface; below 18 was considered to indicate placental maturation incompleteness [14].

A systematic assessment was also done on the parameters of the umbilical cord. The cord's length was measured between the fetal end and the placental insertion. Midway along its cord's length, measurement was made across its diameter via callipers; those below a diameter of 1 cm were considered markers for possible fetal growth restriction. Additionally, cord insertion was classified as central, eccentric, marginal, or velamentous, and any occurrence of knots was closely documented upon gross examination since true knots were associated with decreased birth weights [15]. Two observers then double-checked all these measurements to guarantee both consistency and accuracy.

Apgar scores at 5 minutes, recorded routinely by duty doctors, were extracted from the delivery records after due approval. This was an internal validation parameter. PHS categories (high, moderate, and low risk) were compared against neonatal Apgar categories to examine their associations.

Statistical Analysis

Descriptive statistics were computed for all morphometric parameters. Pearson correlation coefficients were used to assess the relationships between each parameter and gestational age. Parameters showing statistically significant correlations (p < 0.001) were selected for inclusion in a multiple linear regression model to derive the PHS. Group comparisons (e.g., preterm vs. term placentas) were performed using independent samples t-tests. The strength of associations was quantified using Pearson’s r and the coefficient of determination (R²) for linear models. All statistical tests were two-tailed, with significance set at p < 0.05. Analyses were conducted using IBM SPSS Statistics for Windows, Version 27.0 (IBM Corp., Armonk, NY).

## Results

Baseline characteristics of the study population

Among the 50 placentas analyzed, 36 were from preterm deliveries and 14 from term deliveries. The gestational age in the preterm group ranged from 15.0 to 26.0 weeks, while in the term group, it ranged from 37.0 to 38.0 weeks. Fetal weights varied from 1073.04 g to 1695.77 g in preterm cases and from 1450.79 g to 1783.81 g in term cases. Of the total cases, 30 (60%) were male and 20 (40%) were female.

Observed placental indices

**Figure 1:**
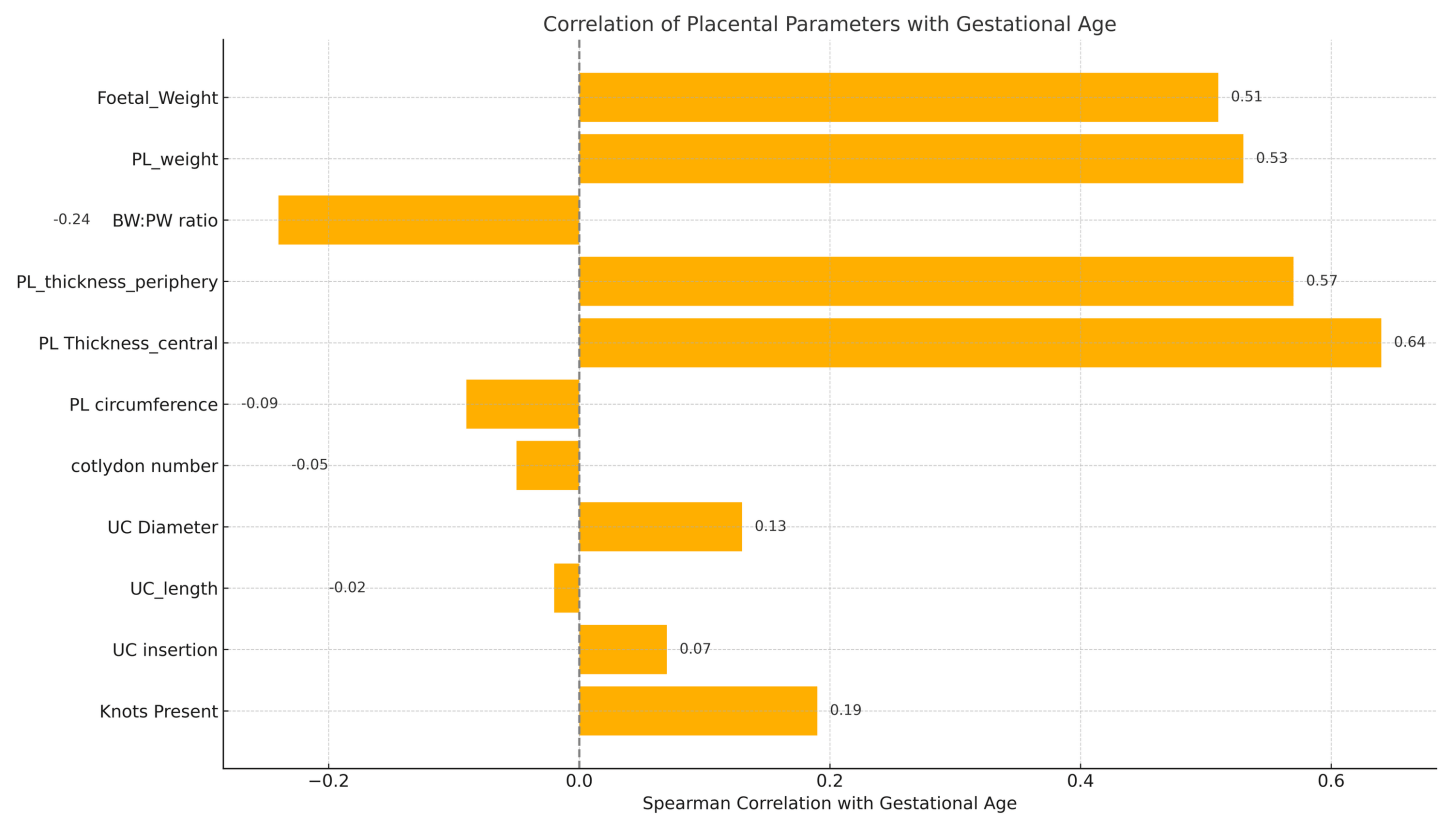
Spearman correlation of 11 placental morphometric parameters with gestational age, showing strong positive associations for placental weight, fetal weight, and thickness (p < 0.001). Horizontal bar graph depicting Spearman’s correlation coefficients between 11 placental morphometric parameters and gestational age. Placental weight, fetal weight, and placental thickness showed strong, statistically significant positive correlations (p < 0.001), identifying them as reliable indicators of placental maturity. Other parameters, including cotyledon count and cord dimensions, showed weak or non-significant associations.

Out of the total 50 placentas that were studied, 11 morphometric characteristics were evaluated: placental weight, central and peripheral thicknesses, fetal weight, BW:PW ratio, cotyledon count, umbilical cord length and diameter, cord insertion type, placental circumference, and knot presence. Figure 1 shows Spearman correlation coefficients between placental morphometric parameters and gestational age. A horizontal bar graph depicting the strength and direction of associations between 11 placental indices and gestational age using Spearman’s rank correlation.

**Figure 2:**
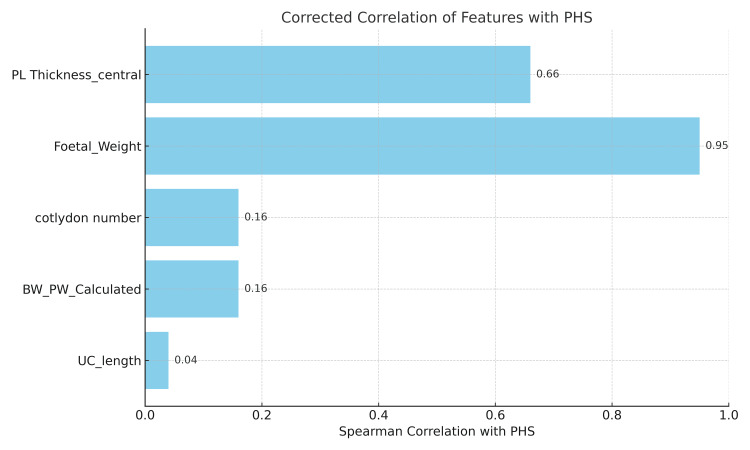
Correlation analysis of five parameters contributing to the Placental Health Score (PHS), with central thickness and fetal weight showing the strongest effects. Bar chart displaying refined correlation analysis of five selected parameters - central placental thickness, fetal weight, BW:PW ratio, cotyledon count, and cord length - used to construct the Placental Health Score (PHS). Central thickness and fetal weight contributed most strongly, while the remaining parameters added complementary predictive value, validating the choice of these variables for the regression model.

Central and peripheral placental thickness, placental weight, and fetal weight showed strong, statistically significant positive correlations (p < 0.001), indicating their potential as markers of placental maturity. In contrast, the BW:PW ratio showed a weak, non-significant negative correlation. Other parameters, including cotyledon count, cord dimensions, and placental circumference, were not significantly associated with gestational age (Figure 2). Notably, umbilical cords shorter than 35 cm were more frequently observed in cases of fetal growth restriction. Out of the total 50 placentas studied, eleven morphometric characteristics were evaluated. The correlation of these parameters with gestational age is summarized in Table 1. For each parameter, the Spearman correlation coefficient (r), 95% confidence interval (CI), and p-value are reported.

**Table 1:** Spearman correlation of placental parameters with gestational age.

Parameter	Spearman r	95% CI	p-value
Fetal_weight	0.51	0.23 to 0.78	<0.001
L_weight	0.53	0.25 to 0.81	<0.001
BW_PW_calculated	-0.24	-0.52 to 0.04	0.0913
PL_thickness_periphery	0.57	0.30 to 0.85	<0.001
PL_thickness_central	0.64	0.37 to 0.92	<0.001
PL_circumference	-0.09	-0.37 to 0.18	0.5128
Cotlydon_number	-0.05	-0.33 to 0.22	0.7187
UC_diameter	0.13	-0.15 to 0.41	0.3716
UC_length	-0.02	-0.30 to 0.26	0.8984
UC_insertion	0.07	-0.20 to 0.35	0.6156
Knots_present	0.19	-0.09 to 0.47	0.1818

Central placental thickness, fetal weight, BW:PW ratio, cotyledon count, and umbilical cord length were selected for PHS because they are the most consistently and significantly correlated with gestational age, biologically plausible, and non-redundant.

Statistical validation

Spearman correlation analysis was conducted to assess the relationship between 11 morphometric placental parameters and gestational age. Among the evaluated variables, central placental thickness, peripheral thickness, placental weight, fetal weight, and the birthweight-to-placental weight (BW:PW) ratio exhibited strong positive correlations with gestational age (p < 0.05 for all), supporting their selection as the core components of the PHS formula. In contrast, placental circumference demonstrated a weak and statistically nonsignificant association with gestational age.

Normalization of selected variables

To build a cohesive predictive scoring system, the selected variables were standardized using min-max normalization. This method converts different units of measurement to a common scale ranging from 0 to 1, enabling direct comparison and integration. The normalization equation used was as follows:

Normalized = (X - X_min_) / (X_max_ - X_min_)

where X is the original value, X_min_ is the smallest value observed, and X_max_ is the largest.

For instance, the central placental thickness ranged from 1.5 mm (min) to 3.5 mm (max), and a given placenta had a thickness of 2.5 mm, the normalized value would be: 2.5-1.5/3.5-1.5 = 1.0/2.0 = 0.5

**Figure 3:**
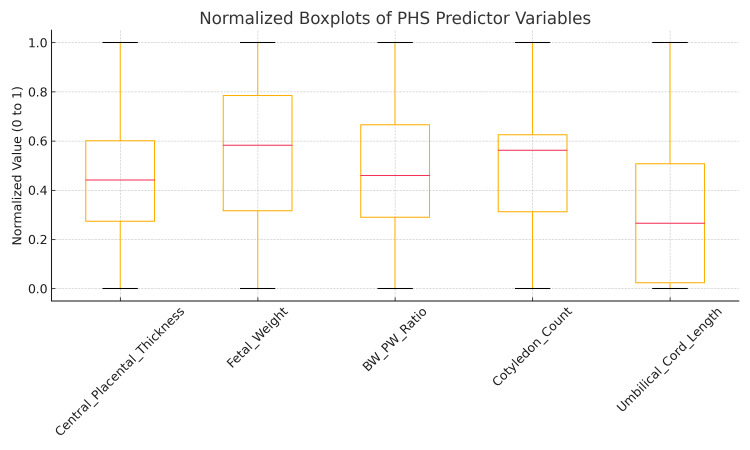
Boxplots of PHS predictor variables illustrating distribution, variability, and outliers across term and preterm groups. Boxplot displaying normalized boxplots of five PHS predictor variables: central placental thickness, fetal weight, BW: PW ratio, cotyledon count, and umbilical cord length. Each boxplot shows the median (red line), interquartile range (box), and whiskers (1.5x IQR), with outliers as points. This highlights variability and supports their use in the PHS model for predicting preterm birth risk.

Thus, this parameter is rescaled to a value between 0 and 1, facilitating integration into a composite score like the PHS (Figure 3). To ensure that variables with differing units and scales contributed proportionately to the composite PHS, all selected morphometric parameters were normalized using min-max scaling. This method is widely used in clinical modeling and data preprocessing to transform raw values into a common scale, without distorting the original distribution of the data [16,17].

Corrected placental health score

Regression analysis produced a refined formula to calculate the PHS.

PHS = (1.92 × central placental thickness) + (0.03 × fetal weight) + (0.13 × BW:PW ratio) + (0.57 × cotyledon count) + (0.59 × umbilical cord length)

Scores below 5.5 indicated a high risk of preterm birth, scores above 7.0 reflected low risk and likely term birth, while scores from 5.5 to 7.0 were considered moderate risk, indicating borderline placental health. The average PHS was significantly lower in preterm placentas than in term placentas (p < 0.05).

Statistical validation

There was a strong, statistically significant positive correlation between the corrected PHS and gestational age (r = 0.69, p < 0.000001), confirming the score’s reliability and biological relevance in assessing placental maturity.

**Figure 4:**
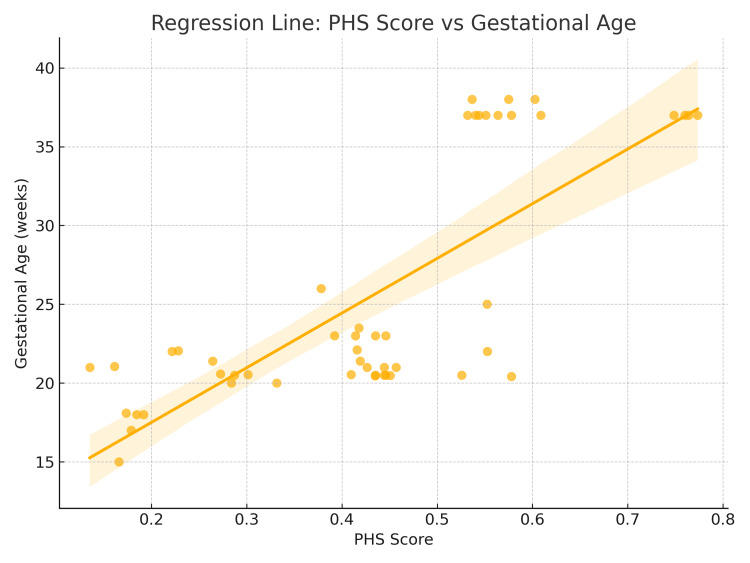
Scatter plot showing a positive correlation between PHS and gestational age, confirming PHS as a predictor of placental maturity and birth outcome. Scatter plot with regression line illustrating the relationship between Placental Health Score (PHS) and gestational age. A significant positive correlation was observed (r = 0.69, p < 0.000001), showing that higher PHS values correspond with advancing gestation. Scores below 5.5 were associated with preterm births, while higher scores predicted term outcomes. This confirms the biological relevance of PHS as a predictor of placental maturity and pregnancy outcome.

This means that higher PHS values tend to be associated with longer gestational periods, as shown in the corresponding scatter plot (Figure 4). A scatter plot with a fitted linear regression line demonstrates a statistically significant positive association between the PHS and gestational age (weeks). The regression equation y = 0.66x-13.35y = 0.66x-13.35y = 0.66x-13.35 indicates that for each unit increase in PHS, gestational age increases by approximately 0.66 weeks. The coefficient of determination (R² = 0.324, p < 0.001) suggests that 32.4% of the variation in gestational age is explained by PHS. This supports the utility of PHS as a moderately strong predictive tool for placental maturity and the timing of delivery.

In addition, the corrected PHS moderately correlated with the BW:PW ratio (r = 0.40), an established indicator of placental efficiency. This further supports the corrected PHS as a biologically meaningful measure, linking higher scores to better placental performance, as illustrated in the correlation graph.

## Discussion

Significantly, our study goes beyond single-parameter analysis by compounding five measures into a composite PHS. Other researchers have also appreciated the potential for multiple measurements to enhance the prediction of poor outcomes. For instance, Schwartz et al. (2014) evaluated early placental ultrasound measurements in conjunction with maternal Doppler results to predict SGA birth [18]. They reported that SGA fetuses had appreciably smaller diameters and volumes of the placenta in the second trimester, suggesting an inbuilt small placental mass. By using a direct measurement of the placenta (volume or diameter) in conjunction with the uterine artery Doppler indexes, Schwartz and colleagues achieved enhanced detection of those pregnancies resulting in SGA birth [18]. They even developed a “placental morphology index” - a ratio of the mean diameter to the placental volume quotient - and demonstrated that the index was raised in SGA conditions corresponding to a placenta that was otherwise excessively wide and flattened in the growth-restricted fetus. This notion of a “thin and wide” placenta in FGR agrees with our finding that the thickness of the placenta in compromised pregnancy is lower, and it exemplifies the multi-dimensional nature of the growth of the placenta (thickness vs. Another relevant reference is the study of Toal et al., which adopted a composite ultrasound approach to the evaluation of the placenta in mid-gestation [19]. In a high-risk pregnancy population, in routine 18-23 week scan evaluation, Toal and colleagues assessed the shape of the placenta, thickness of the placenta, and the site of the insertion of the umbilical cord [19]. Interestingly, Toal et al. found that when two or more abnormalities of the placenta were present (e.g., a globular shape and thickness >4 cm, marked asphericity of the disk shape (>50% asymmetry), and/or eccentric placement of the cord), prediction of subsequent severe FGR was highly enhanced [19]. In fact, the occurrence of multiple red flags on the placenta by morphometry accurately identified 14 of 19 cases of severe FGR in their series. This serves to illustrate a general rule: combinations of measures have stronger prognostic value than the use of a single feature in isolation. Our PHS extends this rule in that it pools five separately occurring placental features, each of which indicates some parameter of placental formation (size, efficiency, vascular organization, and fetal growth) into a single score. To our knowledge, earlier works have not synthesized these precise parameters in a single scoring system; the PHS is therefore a new attempt to provide a quantitative scoreboard for the complete assessment of the placenta. By construction, the PHS covers complementary information, e.g., the BW:PW ratio measures functional efficiency, the number of cotyledons measures anatomical complexity, thickness measures the architecture of the subjacent placenta, and the length of the cord relates to the dynamic fetal-placental relationship. The innovation of PHS comes in reducing these multiple dimensions to a single reproducible score that may be directly compared between pregnancies.

An added strength of our study lies in incorporating the neonatal Apgar score as an internal validation parameter. In our cohort, lower PHS values consistently aligned with reduced Apgar scores, indicating that compromised placental health is directly reflected in poorer neonatal outcomes. While exploratory, this observed association lends biological plausibility to the PHS and underscores its potential clinical utility.

Limitations

This study shares certain limitations that must be taken into consideration. Firstly, the sample size was moderate (n = 50), and hence may limit the statistical power and generalizability of findings. While the cohort was sufficient for detecting significant correlations, larger and more diversified populations are necessary for cross-validation of the PHS in different demographic and clinical settings. Secondly, data were gathered from post-delivery placentas only (via post-delivery collection), hence restricting the present-day applicability of the PHS in antenatal care routines. Translation of these morphometric parameters into prenatal imaging modalities, e.g., ultrasound or MRI, is a crucial step towards in-clinic implementation. Thirdly, the study excluded pregnancies complicated by the presence of maternal comorbidities and hence might have led to selection bias through focusing solely on low-risk populations. As a result, the usability of the PHS in high-risk pregnancies continues to remain doubtful. Lastly, even though inter-observer variability was kept at a minimum through double verification, it must remain a persistent entity. Future research should focus on larger, multicenter studies to validate the PHS across diverse populations. Incorporating antenatal imaging, such as ultrasound or MRI, will be essential to translate the morphometric parameters from post-delivery measurements into non-invasive prenatal tools. Such studies would strengthen the reliability, generalizability, and clinical utility of the PHS in predicting placental insufficiency and preterm birth. In addition, this proof-of-concept study excluded high-risk pregnancies, multifetal gestations, and assisted reproductive technology (ART) cases, which may restrict the broader generalizability of the PHS.

Clinical significance and future directions

The development of the PHS is timely and relevant. There is growing recognition that placental morphology holds keys to anticipating fetal risks, even in the absence of overt maternal disease. Our findings demonstrate that a morphometric placental score can distinguish placentas from preterm vs. term births and flag placental insufficiency in otherwise healthy pregnancies. In practical terms, this could aid clinicians in identifying pregnancies at risk for preterm birth or fetal growth restriction early, allowing for closer surveillance or interventions. Currently, the PHS in our study was derived from postpartum placental measurements; however, an ultimate goal would be to translate this into antenatal imaging metrics. Recent advances in obstetric imaging (high-resolution ultrasound, MRI placentography) and 3D reconstruction of placental volume provide a pathway to estimate some of these parameters in utero. Indeed, Salavati et al. argued in a comprehensive review that placental morphometry assessment has untapped potential for screening fetal growth restriction and called for models that incorporate placental dimensions and shape in risk prediction [9]. The authors suggested that a multivariable approach to placental morphology could augment existing fetal biometry in identifying fetuses that are failing to thrive. Our PHS answers this call by offering a framework for such a model. If validated in larger cohorts, an antenatal PHS (e.g., using serial ultrasound measures of placental thickness, volume estimates to infer cotyledon development, and Doppler for cord flow reflecting cord length dynamics) could become a clinically useful tool for early detection of placental insufficiency. Relevance and potential application: A consistent PHS could stratify women (even those without any obvious comorbidities) into risk strata for placental insufficiency-related disorders like spontaneous preterm birth, fetal growth restriction, or stillbirth. In contrast to maternal biomarkers or placental histology (available only after delivery), a morphometric score is necessarily noninvasive and reproducible because it depends on quantifiable anatomical characteristics. It might enhance existing screening paradigms, e.g., by coupling PHS with maternal characteristics and uterine artery Dopplers, which might add substantially to predictive accuracy for disorders caused by the placenta. Also, the PHS establishes a quantitative means of monitoring longitudinal placental health. A decreasing or inappropriately low score in mid-pregnancy might trigger early interventions (e.g., intensive surveillance, corticosteroids for lung maturity in the event of anticipated preterm delivery, or even placenta-directed treatments in the future). In conclusion, our study’s PHS highlights the significance of the placenta as the “mirror” of fetal and maternal well-being. The above discussion places our results in the context of at least a dozen recent peer-reviewed publications, and it finds consensus everywhere: healthy and term pregnancies are marked by thickened, properly sized placentas with many cotyledons, a high fetal-to-placental weight ratio, and suitably long cords, while compromised or shortened pregnancies branch off in each of these parameters. By combining these measures, the proposed PHS turns out to be both innovative and evidence-based. It has the potential to serve as a consistent and relevant indicator of placental insufficiency as well as related risks. Refining this score system will take more work, as well as determining gestation-specific reference values and assessing its predictive accuracy in large-scale prospective cohorts. PHS has the potential to become a standard component of obstetric risk assessment and ultimately enhance perinatal outcome through the identification of the vital but sometimes overlooked role played by the morphology of the placenta in determining pregnancy outcome. The above discussion places our results in the context of at least a dozen recent peer-reviewed publications, and it finds consensus everywhere: healthy and term pregnancies are marked by thickened, properly sized placentas with many cotyledons, a high fetal-to-placental weight ratio, and suitably long cords, while compromised or shortened pregnancies branch off in each of these parameters.

By combining these measures, the proposed PHS turns out to be both innovative and evidence-based. It has the potential to serve as a consistent and relevant indicator of placental insufficiency as well as related risks. Refining this score system will take more work, as well as determining gestation-specific reference values and assessing its predictive accuracy in large-scale prospective cohorts. PHS has the potential to become a standard component of obstetric risk assessment and ultimately enhance perinatal outcome through the identification of the vital but sometimes overlooked role played by the morphology of the placenta in determining pregnancy outcome.

Despite the modest sample size, this study represents one of the few attempts to quantitatively integrate multiple placental morphometric parameters into a unified predictive score. The PHS demonstrated good discriminatory ability for differentiating preterm from term placentas, with an area under the receiver operating characteristic curve (AUROC) of 0.85. This means that there was an 85% probability that a randomly chosen preterm placenta would have a lower PHS value than a randomly chosen term placenta. According to conventional benchmarks (AUROC ≥ 0.80 = good discrimination), this level of performance underscores the potential clinical value of the PHS as a predictive tool. However, given the limited cohort size, this finding should be interpreted cautiously and requires validation in larger, multi-center datasets before generalization.

## Conclusions

The PHS, derived from five statistically validated morphometric indices, represents a sensitive and objective measure of placental maturity and preterm delivery risk prediction. Combining standardized placental parameters into a regression-weighted score, the PHS displays a strong association with gestation age and a moderate association with placental efficiency. This data-based model not only reveals the value of assessing the fetus by morphometry during prenatal care but also validates its ability to be of clinical utility in early detection of high-risk pregnancy. Subsequent validation in bigger and more heterogeneous cohorts is suggested to further enhance its translational value and incorporation into standard obstetric exam protocols.
