# Correction: Placental Health Score (PHS) for Early Prediction of Placental Insufficiency and Preterm Birth: A Cross-Sectional Analytical Study

**DOI:** 10.7759/cureus.c404

**Published:** 2026-02-20

**Authors:** Mamata Panigrahi, Shweta Solan, Prajnaparamita Samanta

**Affiliations:** 1 Anatomy, Kalinga Institute of Medical Sciences, Bhubaneswar, IND

This article has been corrected to remove figures 1 and 2, which were AI-generated images depicting the placentas and measurement procedures. Per journal policy, the use of AI-generated images is not permitted in published articles. The authors provided the journal with data and images to verify the manuscript's authenticity. 

